# Progress in Surgery

**Published:** 1898-10-29

**Authors:** 


### Progress in Surgery.

### SURGERY OF THE ŒSOPHAGUS AND STOMACH.

(*Continued from page* 51.)

**Gastro-enterostomy**, says Stendel[6] has a 12 per cent. less mortality when performed with the Murphy button than without it. Hahn[7] gives his statistics of 141 cases of gastro-enterostomy and 28 of resection. Of these 28, 18 were cured. When patients are very weak he recommended the operation of resection in two stages—first, the gastro-enterostomy, and after some days the resection. Weir[8] believes it best to combine an entero-enterostomy with the gastro-enterostomy. As regards the latter, he says the anterior operation (Wolfler's), while easier, is associated with dragging effects of the omentum and colon, and also has the disadvantage that may come from any distension of the stomach, whereby not only is the opening raised to a higher level and unduly twisted, but more tension is likewise thus made on the attached intestine. Hence the posterior opening (*v.* Hacker's) is more frequently advised by surgeons. Here by a median incision the stomach is exposed, and the omentum and transverse colon are drawn out from the wound. The mesocolon is now torn or cut through to reach the posterior wall of the stomach over an area some two inches in diameter; through this the stomach is drawn out beyond the wound. One can now, if it is considered desirable, shut off the cavity of the lesser peritoneum, which has thus been opened into, by stitching the torn edge of the mesentery to the stomach, or this can be disregarded. The jejunum is now sought for after Sccin's plan, which is to pull moderately on the colon, after it—with the omentum—has been drawn out of the wound; this stretches its mesentery a little, and near its base will be found, either over the vertebra or to its left, the intestine. Traction upon this will show its duodenal fixation, and then some 30 to 60 centimètres of this bowel are brought out to be attached to the posterior wall of the stomach, eight or ten centimètres from the pylorus. Approximation is made by the Murphy button. The mortality in 26 benign cases collected by Chlumskig was 26 9 per cent., and in 115 malignant cases 38 3 per cent. Weir's conclusions are as follows: (1) That gastro-enterostomy, as usually performed, is yet an unsatisfactory operation; (2) That its principal relievable danger is from bile or stomach retention, due to operative defects, either primary or secondary, *e g*, spurs, kinks, twisted or distorted openings, and, possibly, stomach atony; (3) That a posterior stomach opening favours gravitation by the evacuation of the organ, particularly if it is atonied, and diminishes the risk of pressure or of pulling on the attached intestine; (4) that the operation is best conducted by means of a Murphy button; (5) that a gastro-enterostomy, associated with an entero-anastomosis in the afferent and efferent portions of the jejunum, probably gives the best assurance against obstruction. Experience in this method should, however, be enlarged to ascertain if the increased risk of the additional button over-tops the danger of the bile and pancreatic obstruction. A successful case of gastro-enterostomy by direct suture is reported by Wallis.[9]

**Gastrotomy** on a "human ostrich," aged 22 years, is reported by Meisenbach.[10] The man had been in the habit of swallowing glass, metal, &c., for nine years, and had only recently complained of pain in the stomach. While standing, a mass the size of the hand could be felt in the epigastric and umbilical regions. Gastrotomy was performed, and 118 articles, besides about an ounce of broken glass, and weighing in all one pound, were removed. The articles were: 27 staples, 15 screws (1 in. and 1½ in.), 52 nails (2 in. and 1½ in.), 21 cartridges, two pocket knife blades, and two inches of brass chain. The patient recovered, but an attack of pneumonia of the right base—attributed to the use of the Röntgen rays—followed the operation. Keen[11] advocates exploratory gastrotomy in order to obtain a positive diagnosis when other means have failed. The proceeding is practically innocuous, and is quite justifiable under the circumstances mentioned. In order to prevent any possible septic contamination, he insists on the necessity in this, as in all similar operations on the stomach, of washing out this organ, and, before it is opened, of bringing it out of the abdominal cavity, and of protecting this cavity by a wall of iodoform gauze. In this way the operation can be made a practically extraperitoneal one.

**Gastrorraphy**, or gastroplication, for dilated stomach not depending upon any obvious or removable cause in or around the pylorus or duodenum, is dealt with by Moyniham,[12] who operated upon a case after the manner suggested by Bennett. Twelve sutures at a distance of about an inch from each other were passed, and each suture picked up the stomach wall at nine points. The sutures were made to radiate from the lesser to the greater curvature, and included all the anterior part of the stomach wall, except for a small area at the pyloric and cardiac extremities. The sutures were drawn upon, held up, and tied one by one. It was then seen that there were slight bulgings at each end of the reef which had been made, and a second and third series of sutures were passed, one at each end of the stomach, so as to do away with this irregularity of outline. Special care was taken to round off the pyloric end so that no bulging or kinking would be possible. A median incision was employed. An immediate relief from the severe and disabling symptoms is observed in these cases, but whether such relief is permanent is a question which can only be answered in the future.

**Gastric Ulcer.**—Dieulafoy[13] contends that an operation should be performed in severe cases of hæmatemesis occurring in this disease, and quotes a case where after opening the stomach the bleeding-point was closed with three fine sutures. Twenty days afterwards the patient was completely cured. Hayem[14] and others are not, however, disposed to agree with this treatment, as the diagnosis is difficult, for hæmatemesis may occur in hepatic derangements and in syphilis. Sharkey[15] and Anderson[16] each record successful cases of operation for perforated gastric ulcer. In the latter case no drain was used.

**Pyloroplasty** and posterior gastro-enterostomy are, according to Carle and Fantino[17] the two best operations for non-malignant stricture of the pylorus. Pyloroplasty is dangerous when there is extensive and pronounced thickening, or when peri-pyloritis exists with adhesions to surrounding viscera; for these cases and also for stenosis of the duodenum, gastro-enterostomy or resection is the only possible method. When either operation can be adopted, the authors recommend gastro-enterostomy for the cases (hyposthenic) in which there is a diminution of the motor and secretory power of the stomach, and pyloroplasty in spasmodic strictures and in cicatricial stenosis of an annular form, as this operation preserves the normal relations of the viscera, and is not followed by regurgitation of bile.

**Statistics** of various operations on the stomach are given in a series of articles by Barker[18] and Keen[19]. Both point out the increased mortality produced in perforating gastric ulcer by the lapse of time before operation. The former, quoting from 359 cases of pylorectomy collected by Haberkant, gives the mortality of pylorectomy as 54 per cent. in malignant, and 40 per cent. in non-malignant cases.

[6] New York Med. Rec., May 7, 1898, p. 677. [7] Ibid., p. 678. [8] Ibid., April 16, 1898, p. 541. [9] Brit. Med. Jour.. May 7, 1898, p. 1195. [10] Lancet, June 25, 1898, p. 1767. [11] Brit. Med. Jour., June 11, 1898. [12] Lancet, April 30, 1898, p. 1177. [13] Dublin Jour. Med. Sci., May, 1898, p. 441. [14] Ibid., p. 442. [15] Lancet, May 14, 1898, p. 1323. [16] Ibid. [17] Brit. Med. Jour., May 21, 1898; and Il Policlinico, April 15, 1898. [18] Clin. Jour., May 4, 11, 18, and 25, 1898. [19] New York Med. Jour., May 7 and 21, and June 4 and 11, 1898.

